# Insanity and Its Treatment

**Published:** 1882-01

**Authors:** 


Insanity and Its Treatment. Lectures on the Treatment of Insanity and Kindred Nervous Diseases. By *Samuel Worcester, M. D.*, Lecturer on Insanity, Nervous Diseases and Dermatology, at Boston University School of Medicine, etc., etc. Pp. 462, price $3.50. Boericke & Tafel: New York and Philadelphia. Homœopathic Publishing Co., No. 2 Finsbury Circus: London. 1882.

Dr. Worcester, the author of this new work on insanity, is favorably known to the profession through his "Repertory to the Modalities," a very useful little work. The scope and design of the present work is much more ambitious and difficult. Of all the diseases with which medical men have to deal, those of the mind are the most puzzling, difficult, and, we may add, interesting. It is only recently that any advance has been made in the treatment of the insane. Formerly they were considered to be "possessed" by evil spirits, etc.; the only treatment was to lock them up and place brutal keepers to guard them. In more recent times, it has become well established that the brain has its diseases, as have the nerves and tissues of other parts. Physiology has shown the mind to be most closely connected with the body, to be composed of nerve cells, filaments, etc., to be dependant for its proper action upon the common laws of waste and supply, exhaustion and reparation; beyond this physiology has not yet gone, and pathology is here, at least, a desert waste.

"Its *modus operandi* is, and perhaps always will be, utterly unknown to us. * * * The great pathologist (Virchow), indeed, entertains little hope that any positive knowledge of the *modus operandi* of this apparatus (of cells in the vortex of the cerebrum and cerebellum) will ever be acquired." * Here, then, is an important class of diseases, whose pathology is confessedly crude; for their treatment we must then rely upon the despised symptoms.

This work of Dr. Worcester's comprises a series of lectures delivered at the Boston University School of Medicine, and very good lectures they are. Commencing with a general survey of mental disease—giving definitions,

---

* Bucknill and Tuke, on Psychological Medicine, 3rd edition, p. 486.

early symptoms, causes and classification—the lecturer then gives clear and brief reviews of the various forms of mental disease, such as Dementia. Melancholia, Mania, Folie Circulaire, etc., etc, Indications for the remedies, most often indicated, are clearly given. Yet this part of the work could have been profitably expanded.

